# The Effect of Orexin-A on Cardiac Dysfunction Mediated by NADPH Oxidase-Derived Superoxide Anion in Ventrolateral Medulla

**DOI:** 10.1371/journal.pone.0069840

**Published:** 2013-07-26

**Authors:** Jun Chen, Chunmei Xia, Jin Wang, Meiyan Jiang, Huanhuan Zhang, Chengrong Zhang, Minxia Zhu, Linlin Shen, Danian Zhu

**Affiliations:** 1 Department of Physiology and Pathophysiology, Shanghai Medical College of Fudan University, Shanghai, China; 2 Department of Pathology, Changzheng Hospital, Second Military Medical University, Shanghai, China; 3 Cell Electrophysiology Laboratory, Wannan Medical College, Wuhu, Anhui, China; Virginia Commonwealth University, United States of America

## Abstract

Hypocretin/orexin-producing neurons, located in the perifornical region of the lateral hypothalamus area (LHA) and projecting to the brain sites of rostral ventrolateral medulla (RVLM), involve in the increase of sympathetic activity, thereby regulating cardiovascular function. The current study was designed to test the hypothesis that the central orexin-A (OXA) could be involved in the cardiovascular dysfunction of acute myocardial infarction (AMI) by releasing NAD(P)H oxidase-derived superoxide anion (O_2_^−^) generation in RVLM, AMI rat model established by ligating the left anterior descending (LAD) coronary artery to induce manifestation of cardiac dysfunction, monitored by the indicators as heart rate (HR), heart rate variability (HRV), mean arterial pressure (MAP) and left intraventricular pressure. The results showed that the expressions of OXA in LHA and orexin 1 receptor (OX_1_R) increased in RVLM of AMI rats. The double immunofluorescent staining indicated that OX_1_R positive cells and NAD(P)H oxidative subunit gp91phox or p47phox-immunoreactive (IR) cells were co-localized in RVLM. Microinjection of OXA into the cerebral ventricle significantly increased O_2_^−^ production and mRNA expression of NAD(P)H oxidase subunits when compared with aCSF-treated ones. Exogenous OXA administration in RVLM produced pressor and tachycardiac effects. Furthermore, the antagonist of OX_1_R and OX_2_R (SB-408124 and TCS OX2 29, respectively) or apocynin (APO), an inhibitor of NAD(P)H oxidase, partly abolished those cardiovascular responses of OXA. HRV power spectral analysis showed that exogenous OXA led to decreased HF component of HRV and increased LF/HF ratio in comparison with aCSF, which suggested that OXA might be related to sympathovagal imbalance. As indicated by the results, OXA might participate in the central regulation of cardiovascular activities by disturbing the sympathovagal balance in AMI, which could be explained by the possibility that OXR and NAD(P)H-derived O_2_^−^ in RVLM mediates OXA-induced cardiovascular responses.

## Introduction

Studies have revealed that Hypocretin/orexin (OX)-producing neurons, located in the perifornical region of the lateral hypothalamus, project to brain sites important in behavioral [1], [2], cardiovascular [3], [4] and endocrine function [4], [5]. The orexin family includes OXA and OXB, which is formed from a single precursor prepro-orexin (PPO). OX_1_R and OX_2_R, two binding proteins, mediate the actions of OX [6]. Although orexin-producing neurons are localized to the lateral hypothalamic and perifornical nucleus, OX-containing nerve terminals and receptors are widely distributed throughout the brain, including the brainstem area that includes the RVLM [7]–[9]. Although they have many pharmacological effects in common, OXA and OXB present some actions unique to each other [10]. Ample evidence has shown that intracisternal- and RVLM-injected OX evoke a rise in blood pressure (BP) and HR in anesthetized rats [11], with OXA being made more effective. It was reported that OXA, when administered through an intracerebroventricular [12], [13], or intrathecal [14] injections, increased BP, HR, and sympathetic activity in rats and rabbits. Injections of OXA in the vasopressor region of the RVLM depolarized vasopressor neurons [15]. The data have indicated that RVLM, a key neural structure involved in mediating the sympathetic activity of OXA in the brain, plays an important role in regulating cardiovascular function.

In both human patients and experimental animal models, myocardial infarction has been shown to be associated with an increased sympathetic drive [16]. Sympathetic drive is important in relation to morbidity and mortality after acute myocardial infarction [17]. Our previous study had showed that OXA administrated into the RVLM evoked the pressor response via OX receptor-dependent mechanism, on which both OX_1_R and OX_2_R produced significant effects [18]. OXA was reported to accelerate cardiovascular response by activating sympatho-excitatory neurons in the RVLM, which is also an essential part of the central baroreflex pathway pertinent to cardiovascular activity [11], [19]. However, the role of OXA in AMI remains unknown.

Reactive oxygen species (ROS) have been shown to play an important role in various physiological and pathophysiological processes [20]. Evidence has indicated that ROS in paraventricular nucleus (PVN) modulates cardiac sympathetic afferent reflex (CSAR) in rats [21]. In addition, ROS has reported to be linked to the regulation of sympathetic nerve activity in RVLM [22]. In RVLM, NAD(P)H oxidase-derived superoxide anion (O_2_^−^) was found to be necessary for enhanced CSAR response caused by Ang II in the PVN [23], and involved in the central sympathoexcitation of myocardial infarction-induced heart failure [24]. However, there is a dearth of literature on the role of heightened central sympathetic activity due to OXA and NAD(P)H oxidase-derived O_2_^−^ in RVLM as well as on the concurrent pathophysiologic effect upon AMI.

Therefore, the aim of the present study was to identify the effects of centrally-administered OXA on sympathetic activity and cardiac function, and to unravel whether they were mediated by OX receptors. Furthermore, it was intended to determine whether central OXA microinjection would stimulate NAD(P)H oxidase-derived O_2_^−^ production in RVLM. The results showed that centrally-administered OXA might exert an effect on sympathetic outflow activation via both OX_1_R and OX_2_R mechanism, thereby impairing cardiac function by stimulating NAD(P)H oxidase-generated O_2_^−^ in RVLM.

## Materials and Methods

### Ethics Statement

The current experiments conformed to the Regulations for the Administration of Affairs Concerning Experimental Animals, National Committee of Science and Technology of China and Instructive Notions with Respect to Caring for Laboratory Animals, the Ministry of Science and Technology of China, and were approved by the Ethics Committee for Experimental Research, Shanghai Medical College of Fudan University.

### Animal Preparations

Adult Sprague-Dawley male rats weighing 250–300g were housed on a 12-h light/dark cycle with food and water ad libitum and room temperature maintained at 23–24°C. For the experiments, the rats were randomly divided into control (sham-operated) and AMI groups.

### Surgical Procedure

An AMI model was established by ligating the LAD coronary artery in anesthetized rats as previously described [25]. Under pentobarbital anesthesia, the rats were fixed in the supine position on a heated operating platform (H-KWDY; Quanshui Experimental Instrument, Chengdu, China) to maintain body temperature at 37.5±0.5°C by monitoring the rectal temperature. They were incubated and mechanically ventilated with a respirator (DHX-50; Chengdu Instrument Company, Chengdu, China). Physiological arterial blood gas levels were analyzed (Medica Easy Blood Gas Analyser; Medica, Bedford, MA, USA) and maintained by adjusting the respirator and oxygen supplement. The hearts were exposed via left thoracotomy, and a coronary snare was prepared around the left anterior artery. In AMI groups, the left anterior descending coronary artery was ligatured between the pulmonary cone and the left atrial appendage leading to a temporary whitening of the LAD coronary artery blood supply region became white. Subsequently, the rats were intraperitoneally injected with 4.0×10^6^ U of penicillin to prevent infection, and then they were revived at 30°C, while the control group consisted of sham-operated rats (Sham) which only received thoracotomy without coronary artery ligation. At the end of the five-day period, after LAD ligation surgery, the rats underwent hemodynamic measurements by cardiac catheterization prior to being sacrificed and tissue collection.

### Pathophysilogical Examination of AMI Heart

**Figure 1:**
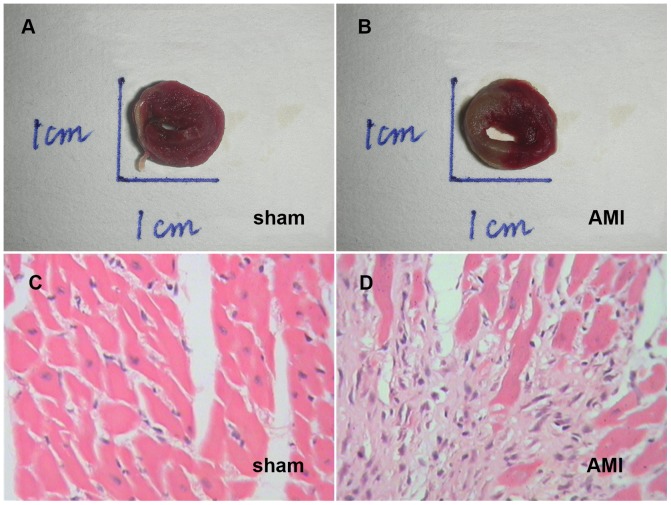
Pathohistological manifestations of AMI heart. TTC staining showing that the viable myocardium stained brick red (A) and the infarct appeared as pale white (B); Hematoxylin-eosin (HE) stain showing the microstructure of myocardium in control (C) and AMI (D) rats. C and D; scale bar = 10 µm.

For MI size identification, 2, 3, 5-triphenyltetrazolium chloride (TTC) was exploited to identify the viable tissue [25]. The heart was transversely sectioned into five sections, with one being made at the site of the ligature. All sections were immediately rinsed in 1% TTC, incubated at 37°C for 30min, and thereafter preserved in 10% formaldehyde for further image analysis. The viable myocardium was stained in brick red, and the infarcts appeared pale white (Fig. 1A). The hearts of some AMI rats were fixed in 4% paraformaldehyde for 48h, then dehydrated in alcohol and embedded in the paraffin according to the routine histological procedures. The sections of the heart tissues (6µm) were prepared to be stained in Harris hematoxylin and Eosin (HE) Solution.

### Immunohistochemistry

The rats were anesthetized to be perfused through the ascending aorta with 200ml heparinized saline, followed by an addition of 200ml freshly prepared 4% paraformaldehyde in 0.1M phosphate buffer saline (PBS, pH7.4). Afterwards, the brain was removed from the skull and post-fixed at 4°C overnight. The fixed brain was placed in 20% sucrose until the brain sank to the bottom, and was then placed in 30% sucrose and 4% paraformaldehyde at 4°C overnight. The coronal sections of the brain (30µm) were made with a cryostat microtome (LeicaCM1900, Germany). A conventional avidin-biotin-peroxidase complex (ABC) technique was performed for immunohistochemical staining [18]. All experiments were conducted at room temperature. Free-floating brain slices were washed in 0.01M PBS (pH 7.4) and incubated in 0.01M PBS containing 0.3% Triton X-100 for 30min. Rinsed in PBS thrice, they were rinsed with 0.3% hydrogen peroxidase/PBS to inactivate the endogenous peroxidase for 10min. And then they were rinsed in PBS thrice to be placed in blocking solution for 20min. The commercially available rabbit antibody against OXA (1∶1000) or goat against OX_1_R (1∶300) was used to stain one set of free-floating sections for OXA and OX_1_R for each rat. The sections were incubated at 37°C for two hours, and incubated at 4°C overnight with the diluted primary antibody. Rinsed with PBS, they were subjected to anti-rabbit or anti-goat IgG incubation and subsequently to ABC complex. The 3, 3-diaminobenzidine (DAB) was used to visualize immunostaining.

The sections were examined under a light microscopy to identify OXA and OX_1_R positive cells in LHA and RVLM, the regions of the hypothalamus and RVLM identified based on the rat brain stereotaxic atlas of Paxinos and Watson [26]. The staining density of OXA-IR neurons in LHA and OX_1_R-IR neurons in RVLM were evaluated as the absolute numbers and relative optical density (ROD), which was calculated based on the previously reported formula (OD_OXA_-OD_background_)/OD_background_ [27]. The image analysis of OXA-IR and OX_1_R-IR neuron number and ROD was performed using Image Measurement Version 1.00 (Department of Physiology and Pathophysiology, Shanghai Medical College of Fudan University, China).

### Double Immunofluorescence Staining

The rats were processed for double immunofluorescence staining to find out whether OX_1_R immunoreactive (IR) cells were co-localized with NAD(P)H oxidase subunits gp91phox or p47phox-IR cells in RVLM. The free-floating sections of the medulla oblongata were simultaneously incubated with two primary antibodies, a goat polyclonal anti-OX_1_R (1∶300) together with a mouse polyclonal anti-gp91phox (1∶100), or with a mouse polyclonal anti-p47phox (1∶100). The same sections were concurrently incubated with two appropriate secondary antibodies (1∶100), donkey anti-goat IgG conjugated with cy3 for OX_1_R, and rabbit anti-mouse IgG conjugated with FITC for gp91phox or p47phox. The processed sections were examined under a Leica DM IRB microscope equipped with Leica C-plan optics. Photomicrographs were taken with a Leica DC-300F digital camera using IM50 software for JPEG images. The sections were viewed under the laser scanning confocal microscope (Leica Microsystems, Bensheim, Germany).

### Real-time PCR Analysis for Prepro-orexin (PPO) mRNA Expression

The tissues of hypothalamus or RVLM were dissected and homogenized in Trizol reagent. RNA was extracted and reverse-transcribed into first-strand cDNA using a cDNA synthesis kit. The mRNA expression of target genes was normalized against that of GAPDH. For PPO gene, the forward and reverse primers were 5′-TCAGACTCCTTGGGTATTTGG-3′ and 5′-GCCCAGGGAACCTTTGTAG-3′, respectively. For GAPDH gene, the forward and reverse primers were 5′-CCCTTCATTGACCTCAACTACATG-3′ and 5′-CTTCTCCATGGTGGTGAAGAC-3′. Two-step real-time PCR denaturing, annealing and extension reactions were performed for 45 cycles of 30 seconds at 95°C and 1min at 58°C in PPO and GAPDH. For gp91phox gene, the forward and reverse primers were 5′-CCAAGATGCCTGGAAACTACC-3′ and 5′-CCCACTAACATCACCACCTCA-3′. For p47phox gene, the forward and reverse primers were 5′-TGGGACTGCCCGTGAAGAT -3′ and 5′-GGATGATGGGACCCGTGAT-3′. PCR amplification was performed as follows: 1 cycle at 95°C for 4 min, followed by 40 cycles of 95°C for 15 s, 60°C for 20 s, 72°C for 20s and 78°C for 20s.

Each sample was analyzed in triplicate, the Ct values for PPO, gp91phox and p47phox subtracted from those of GAPDH to yield △Ct values, the average △Ct calculated for the control group and subtracted from the △Ct of all other samples (including the control group). This resulted in a △△Ct value for all samples, which was then used to calculate the fold changes in PPO, gp91phox and p47phox mRNA levels using the formula 2^–△△Ct^, as recommended by the manufacturer (Bio-Rad Hercules, CA, USA) [28].

### Western Blot Analysis

The tissue samples of RVLM OX_1_R were subjected to Western blot analysis as before [18], [29]. A total protein extract was prepared by homogenizing RVLM tissue in lysis buffer in the presence of protease inhibitor. The total protein concentration was measured via the bicinchoninic acid (BCA) assay. Protein samples of 60µg were separated with 10% sodium dodecyl sulphate–polyacrylamide gel electrophoresis, transferred to polyvinylidene diﬂuoride membranes (Millipore, MA, USA), blocked with 5% non-fat milk and incubated with rabbit polyclonal antibodies against OX_1_R (1∶1000; Millipore, MA, USA) in 5% non-fat milk at 4°C overnight. Mouse polyclonal antibodies against β-actin (1∶3000; Beyotime Institute of Biotechnology, Haimen, China) were incubated as an internal standard. Peroxidase-conjugated anti-rabbit (1∶3000) or anti-mouse (1∶3000) antibodies were used as the secondary antibody. The membranes were probed using an ECL-Plus detection kit on film (Beyotime Institute of Biotechnology, China), and the films, scanned with a photoscanner and analyzed with gel-pro Analyser (Shanghai FURI Science & Technology, Shanghai, China).

### RVLM Microinjection and Hemodynamic Measurements

Five days later after LAD ligation, RVLM microinjection and hemodynamic measurements were made, as previously described [18], [29], [30], before the rats were sacrificed. The rate was anaesthetized with 7ml/kg i.p. injection of composite anaesthetic agent (140g urethane, 7g chloralose and 7g borax per 1L normal saline). The trachea was incubated with a polyethylene tube so that the rat breathed the room air spontaneously. The head was fixed on a stereotaxic apparatus, flexed to an angle of about 45°. The occipital bone was carefully removed to expose the fourth ventricle, with its floor kept at a horizontal level. A stainless steel cannula with the outward diameter of 200µm was inserted into RVLM (1.5–1.9mm ahead of the obex, 1.5–2.0mm right to the midline and 6.6–7.0mm deep from the dorsal surface of the cerebellum) according to the atlas of Paxinos and Watson [26]. One arterial catheter was inserted into the left femoral artery for BP measurement, and the other, inserted into the left ventricle of the heart through the right carotid artery for recording the left intraventricular pressure, using two separate pressure transducers. The two catheters were then connected to a bioelectric signal-processing system (Model SMUP-E, Department of Physiology and Pathophysiology, Shanghai Medical College of Fudan University), from which the data related to cardiac function were obtained for analysis. The analyzed parameters of cardiac function included HR, MAP (mean arterial pressure), left ventricular end-diastolic pressure (LVEDP), maximum positive rate of developed left ventricular pressure (+LVdp/dtmax) and maximum negative rate of developed left ventricular pressure (-LVdp/dtmax). The values of +LVdp/dtmax and −LVdp/dtmax were obtained from the time derivative of the left ventricular pressure in a cardiac cycle.

L-glutamate (2nmol/100nl) was microinjected into the RVLM to preliminarily test whether the needle tip was precisely located in RVLM by an elevation of MAP (ΔMAP≥20mmHg) and HR (ΔHR ≥30bpm). In all animals, a period of 30min was allowed at the beginning of the experiment for MAP and HR stabilization at baseline before treatment.

During the experiment, body temperature was measured with a rectal thermometer and maintained at 37.5±0.5°C using a temperature controller (H-KWDY, Quanshui Experimental Instrument, China). Arterial blood PaO_2_ and PaCO_2_ were periodically monitored through a blood gas analyzer (Medica Easy Blood, Medica, USA), and maintained within normal limits. Arterial blood pH was maintained between 7.35 and 7.45. At the end of the experiment, 2% Pontamine Blue Dye (100nl) was microinjected to confirm the accurate injection sites within RVLM. The animal was scarified by overdose i.p. injection of composite anesthetic agent so that the brain was removed. The rat brain fixed in 10% formalin for 7 days, the frozen brain cross-sections (30µm) were made and stained with 1% Neutral Red to identify the sites of microinjection [29]. The location of each site was identified (Figure S1) and mapped on diagrams of the rat brain according to the atlas of Paxinos and Watson [27].

### Recording and Power Spectral Analysis of HRV

HRV spectral analysis, an established method to quantify the activity of the autonomic nervous system in both humans [31] and animals [32], was conducted. Prior to and after microinjection of test drugs or vehicle, on-line and real-time power spectral analyses of HR signals were simultaneously made using MAP and HR records. Power spectral analysis of HRV was quantified by computing the power spectrum density of the low frequency (LF; 0.25–0.8Hz) and high frequency (HF; 0.8–3Hz) components, as well as the LF /HF ratio, which was continuously displayed during the experiment [29].

### Measurements of O_2_^−^ Production in RVLM

O_2_^−^ production was measured with lucigenin-enhanced chemiluminescence according to the previously described methods [33]; total protein concentration, determined by the BCA assay; ventrolateral medulla, homogenized in a 20mM sodium phosphate buffer (PBS, pH 7.4), containing 0.01mM EDTA by a glass-to-glass homogenizer; the homogenate, subjected to a low speed centrifugation at 1000*g* for 10 minutes at 4°C to remove the nuclei and unbroken cell debris; the pellet, discarded to obtain the supernatant for O_2_^−^ measurement.

In the buffer (2ml) containing lucigenin (5µmol/L), the background chemiluminescence was measured for 5 minutes. This concentration of lucigenin did not appear to be involved in redox cycling and detected O_2_^−^. An aliquot of 100µL of supernatant was then added, and the chemiluminescence was measured for 30 minutes at room temperature (Sirius Luminometer, Berthold, Germany). The O_2_^−^ production was calculated and expressed as mean light unit per min per mg protein.

### Experimental Reagents

The reagents and detection kits were administered as follows: L-glutamate, OXA, N-(6, 8-difluoro-2-methyl-4-quinolinyl)-N0-[4-(dimethylamino) phenyl] urea (SB-408124), 2, 3, 5-triphenyltetrazolium chloride (TTC), rabbit polyclonal antibodies against OXA (1∶1000), goat polyclonal anti-OX1R (1∶300), mouse polyclonal anti-gp91phox (1∶100), and mouse polyclonal anti-p47phox (1∶100) (Sigma-Aldrich, St Louis, Mo, USA); (2S)-1-(3,4-dihydro-6,7-dimethoxy-2(1H)-isoquinolinyl)-3,3-dimethyl-2-[(4-pyridinylmethyl)amino]-1-butanone hydrochloride (TCS OX2 29) (Tocris, Minneapolis, MN, USA); Cy3-labeled donkey anti-goat IgG, Fluorescein isothiocyanate (FITC)-labeled donkey anti-rabbit IgG, mouse polyclonal antibodies against beta-actin and ECL-Plus detection kit (Beyotime Institute of Biotechnology, Haimen, Jiangsu, China); anti-rabbit IgG, avidin-biotin-peroxidase complex (ABC) and 3, 3-diaminobenzidine (DAB) (Shanghai Shenhang Bio-Tech Co., Ltd., Shanghai, China).

The chemicals microinjected into RVLM included L-glutamate (2nmol) dissolved in the artificial cerebrospinal fluid (aCSF, pH 7.4, composition in mM: NaCl 130, KCl 2.99, CaCl_2_ 0.98, MgCl_2_ **^.^**6H_2_O 0.80, NaHCO_3_ 25, Na_2_HPO_4_ **^.^**12H_2_O 0.039, NaH_2_PO4.2H_2_O 0.46); SB-408124 (100pmol), TCS OX2 29 (100pmol) and APO (100pmol) dissolved in 1% dimethyl sulfoxide (DMSO) in aCSF. All injections were made at 100nl within 1min. The dose of OXA was based on our previous study [18].

### Statistical Analysis

Data were expressed as mean±S.E.M. Statistical analysis was performed with the unpaired Student’s *t* test (for sham-control and AMI group comparisons) or one-way ANOVA with Dunnett’s test (for the vehicle-control group and the drug groups comparisons). Differences with *P* less than 0.05 were considered significant.

## Results

### Manifestation with Acute Myocardial Infarction Following LAD Ligation

#### Disordered hemodynamic parameters

Five days later after ligation, the hearts of AMI rates exhibited a kind of dysfunction that was reflected as a significantly increased LVEDP, a decreased +LVdp/dtmax and −LVdp/dtmax when compared with those of sham ones. The hemodynamic parameters of both indicated cardiac dysfunction (Table 1).

**Table 1:** Hemodynamic parameters in control and AMI rats at day 5 post-ligation.

	Control	AMI
HR(bmp)	407.13±9.31	444.67±9.16*
LVEDP(mmHg)	−0.45±0.64	20.81±3.81**
+dp/dt.max(mmHg/s)	6150.14±232.19	3031.96±311.18**
−dp/dt.max(mmHg/s)	−4994.41±203.16	−2612.88±165.48**
MAP(mmHg)	97.04±1.99	80.07±1.73**

LVEDP: left ventricular end-diastolic pressure; +LVdp/dtmax: maximum a positive rate of developed left ventricular pressure; −LVdp/dtmax: maximum a negative rate of developed left ventricular pressure; Values as mean+S.E.M (n = 6).

** *P*<0.05,

* *P*<0.01 when compared with the control group.

HRV power spectral analysis showed that HF component of HRV in AMI group was significantly reduced and LF/HF ratio was increased when compared with that of the control group (Table 2).

**Table 2:** Heart rate variability (HRV) analysis in rat with different treatment.

	LF.rri(nu)	HF.rri(nu)	LF/HF
Control	10.8±3.0	35.7±2.9	0.30±0.02
AMI	12.2±1.9	22.9±2.2**	0.55±0.03**
AMI+aCSF	14.3±0.8	24.2±1.5	0.59±0.02
AMI+OXA	17.0±1.0##	18.5±1.7#	0.93±0.05##
AMI+SB	11.5±0.7	28.3±1.0#	0.40±0.02##
AMI+SB+OXA	15.2±0.6★	21.2±1.2	0.72±0.03★★
AMI+TCS	11.8±0.6	29.2±1.5#	0.42±0.04##
AMI+TCS+OXA	16.4±0.3★★	21.9±1.1	0.76±0.03★★
AMI+APO	11.2±0.5	28.6±0.9#	0.39±0.02##
AMI+APO+OXA	14.7±0.6	21.8±1.2	0.68±0.02★★

Low- (LF) and high-frequency (HF) components of HRV and LF/HF ratio after microinjection of artificial cerebrospinal fluid (aCSF), OXA, SB408124(SB), TCS OX2 29(TCS) or apocynin(APO) in the rostral ventrolateral medulla (RVLM); Values as mean+S.E.M (n = 6);

** *P*<0.01 when compared with the control group;

# *P*<0.05,

## *P*<0.01 when compared with the AMI+aCSF group;

★ *P*<0.05,

★★ *P*<0.01 when compared with the AMI+OXA group.

#### Heart pathological changes

TTC staining showed that the viable myocardium was stained in brick red and the infarct appeared pale white in AMI heart (Fig. 1B). The infarction area was 32.12±1.5% in AMI group, while no infarction was present in the control group (Fig. 1A). Microscopic histology revealed that the non-infarcted myocardium was characterized by an organized pattern in the control group, showing a normal architecture (Fig. 1C). AMI group showed severe myocardial membrane damage, edema and infiltration of inflammatory cells in comparison with the control. Significant myonecrosis with fibroblastic proliferation and presence of inflammatory cells were observed (Fig. 1D).

### Increased Expression of OXA in AMI rats’ Hypothalamus

**Figure 2:**
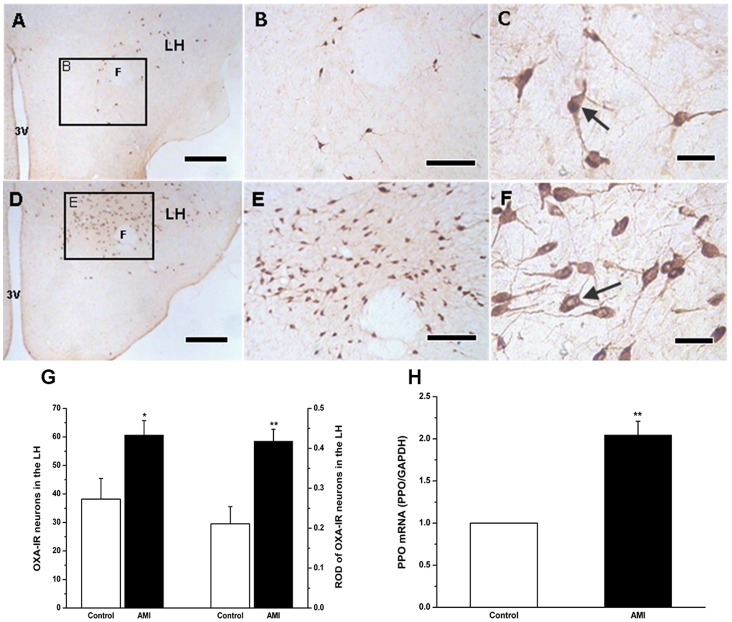
Expression of OXA in LHA of the control and AMI group. A to C showing OXA-IR expressed in control group and D to F showing the AMI group, respectively; the positive cellular morphology indicated by arrows; the number and relative optical density (ROD) of OXA-IR neurons in AMI increased markedly as compared with those of the control rats(G); mRNA level of PPO in the hypothalamus in AMI group increased (H); values as mean±S.E.M (n = 7); * *P*<0.05, **P<0.01 when compared with the control group; scale bars = 50µm in A and D; 20µm in B and E; 10µm in C and F LH: lateral hypothalamic area; F: fornix.

Immunohistochemistry staining showed the OXA-containing neurons located in LHA in both control and AMI group (Fig. 2A–F). The number of OXA-IR neurons (61±5 vs. 38±7, *P*<0.05) (Fig. 2G) and ROD of OXA (0.42±0.03 vs. 0.21±0.04, *P*<0.01) (Fig. 2G) were significantly greater in AMI than in the control (1.5- to 2-fold increase). Furthermore, real-time PCR analysis showed that mRNA expression of prepro-Orexin (PPO) was 2-fold greater in LHA of AMI group than that of the control (Fig. 2H).

### Increased Expression of OX1R in AMI Rats’ RVLM

**Figure 3:**
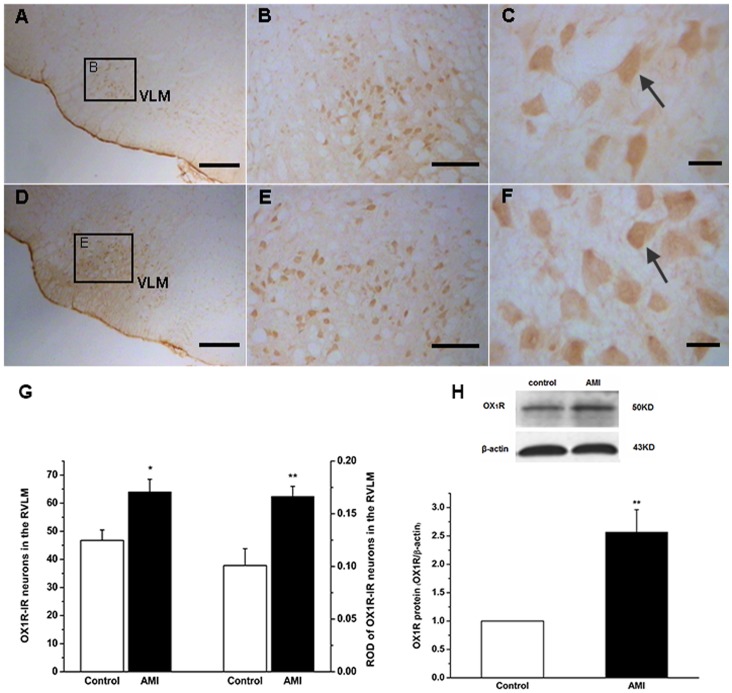
Expression of OX_1_R in RVLM of the control and AMI group. A to C showing OX_1_R expressed in the control group; D to F showing the OX_1_R-IR expressed in AMI group; the positive cellular morphology indicated by arrows; the number and relative optical density (ROD) of OX_1_R -IR neurons in AMI increased significantly when compared with those of the controls (G) (n = 7); Western blot showing the change of OX_1_R expressions in RVLM in the control and AMI group (H) (n  = 4); values as mean+S.E.M; * *P<*0.05, ** *P<*0.01 when compared with the control group; scale bars = 50µm in A and D; 20µm in B and E; 10µm in C and F VLM: ventrolateral medulla.

Immunohistochemistry staining showed that OX_1_R -containing neurons were located in RVLM of both control and AMI group (Fig. 3A–F), the number of OX_1_R -IR neurons (64±4 vs. 47±4, *P*<0.05) (Fig. 3G) and the ROD of OX_1_R expression (0.17±0.01 vs. 0.1±0.02, *P*<0.01) (Fig. 3G) were significantly greater in AMI rats than in the controls. Furthermore, western blot analysis showed a specific band in the gel (Fig. 3H) OX_1_R protein expression 2.5-fold greater in RVLM of AMI group than in the control (Fig. 3H).

### Effect of SB-408124 or TCS OX2 29 on OXA-induced Cardiovascular Responses and HRV

**Figure 4:**
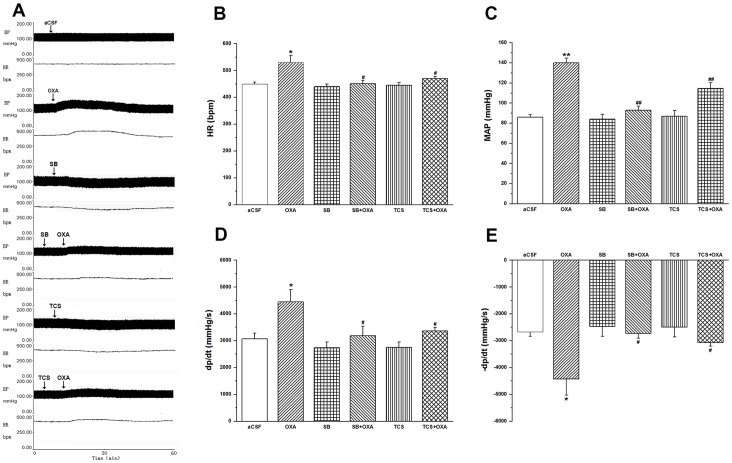
Effects of SB-408124 or TCS OX2 29 on OXA-induced cardiovascular responses in AMI rats. Exogenously administrated OXA into the RVLM evoking pressor and tachycardiac responses; SB-408124 (100 pmol/100 nl) or TCS OX2 29 (100 pmol/100 nl) followed by OXA (100 pmol) partly abolishing the pressor and tachycardiac responses of exogenously administrated OXA into the RVLM; values as means ± S.E.M, n = 7; *P<0.05, **P<0.01 when compared with aCSF group; ^#^ *P*<0.05, ^##^ *P*<0.01 when compared with OXA group.

Microinjection of aCSF into the unilateral RVLM produced a small change in MAP and HR, and that of exogenous OXA (100pmol) into the unilateral RVLM of both AMI rats and the control (Figure S3) caused a significant increase in HR (OXA vs. aCSF: 530±27 vs. 449±7 bpm, *P*<0.05) (Fig. 4A and B), MAP (OXA vs. aCSF: 140±4.7 vs. 86±2.4 mmHg, *P*<0.01) (Fig. 4A and C), +LVdp/dtmax (OXA vs. aCSF: 4446±467 vs. 3066±220mmHg/s, *P*<0.05) and −LVdp/dtmax (OXA vs. aCSF: −4428±602 vs. −2669±169mmHg/s g, *P*<0.05) (Fig. 4D and E). A single microinjection of SB-408124 (100pmol), a selective antagonist of OX_1_R, into the unilateral RVLM of AMI rats produced no obvious effect on MAP and HR. Pre-microinjection of SB-408124 followed by OXA mostly blocked the left ventricle response induced by OXA alone (+LVdp/dtmax: 3183±360 vs. 4446±467 mmHg/s; −LVdp/dtmax: −2725±177 vs. −4428±602 mmHg/s; *P*<0.05) (Fig. 4D and E). From the investigation on whether the OXA-induced cardiovascular responses were also mediated by OX_2_R, the results showed that microinjection of TCS OX2 29 (100pmol) alone into RVLM of AMI rats generated no obvious influence on cardiovascular response, but partially blocked hypertension (114.5±5.6 vs.140±4.7 mmHg; *P*<0.01) and tachycardia (470±8 vs.530±27 bpm; *P*<0.05), as well as the left ventricle response (+LVdp/dtmax: 3364±131 vs. 4446±467 mmHg/s; −LVdp/dtmax: −3074±129 vs. −4428±602 mmHg/s; *P*<0.05), which were all evoked by OXA alone (Fig. 4A–E). The effect of SB-408124 was found to be greater than that of TCS OX2 29.

HRV power spectral analysis showed that in AMI group OXA caused a decrease in HF component of HRV (18.5±1.7 vs. 24.2±1.5; *P*<0.05) and an increase in LF/HF ratio (0.93±0.05 vs. 0.59±0.02; *P*<0.01), when compared with that of aCSF-treated one. Microinjection of SB-408124 or TCS OX2 29 resulted in an increase in HF (28.3±1.0 vs. 24.2±1.5; 29.2±1.5 vs. 24.2±1.5; *P*<0.05) and a decrease in LF/HF ratio (0.4±0.02 vs. 0.59±0.02; 0.42±0.04 vs. 0.59±0.02; *P*<0.01), when compared with that of the aCSF-treated group. Microinjection of SB-408124 or TCS OX2 29 (100pmol) followed by OXA partially blocked the changes of LF/HF ratio induced by OXA alone (0.72±0.03 vs. 0.93±0.05; 0.76±0.02 vs. 0.93±0.05; *P*<0.01) (Table 2).

### Co-localization of OX1R with NAD(P)H Subunits gp91phox or p47phox in the RVLM

**Figure 5:**
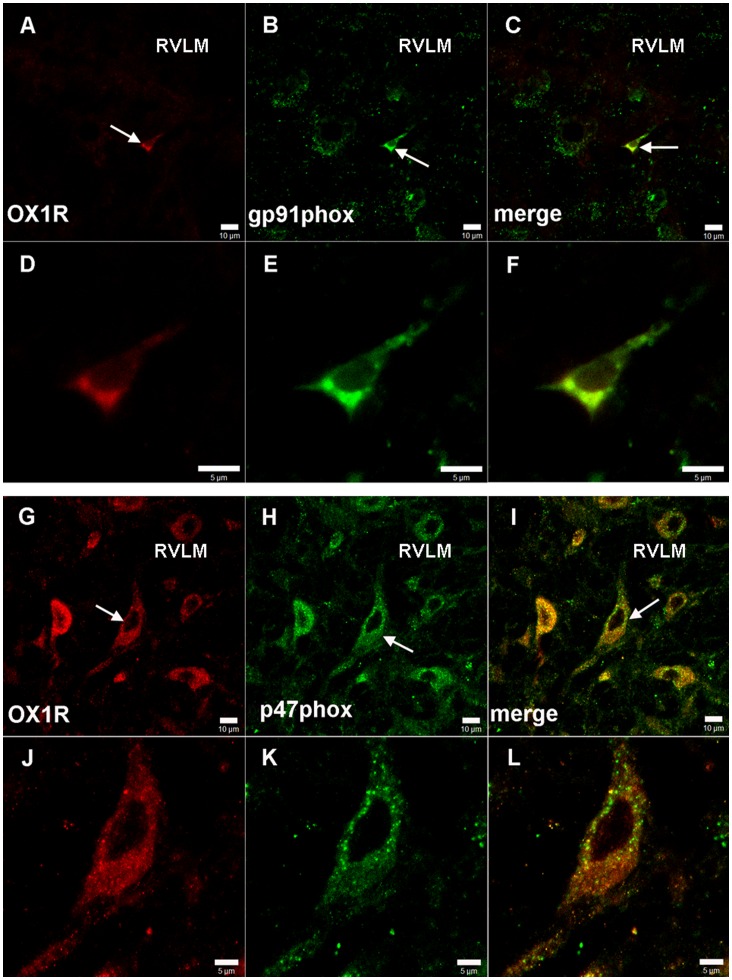
Co-localization of OX_1_R and NADPH oxidase subunits in RVLM. Double immunofluorescent staining showing the OX_1_R and NADPH oxidase subunit gp91^ phox^ or p47 ^phox^ co-localized in RVLM; the OX_1_R shown in red (A, D, G and J), and the gp91 ^phox^ (B and E) or p47 ^phox^ (H and K), in green; the merged images showing yellow color (C, F, I and L); scale bars = 10µm in A, B, C, G, H and I, 5µm in D, E, F, J, k and L. RVLM: rostral ventrolateral medulla.

Double immunofluorescent staining was used to find out whether OX_1_R immunoreactive (OX_1_R-IR) were (Fig. 5A, D, G and J) co-localized with NAD(P)H oxidase subunits gp91phox (Fig. 5B and E) or gp47phox-IR (Fig. 5H and K) in RVLM. OX_1_R -IR cells were observed throughout the confines of RVLM, and found to be co-localized with gp91phox -IR cells by about 80% (Fig. 5C and F), and with p47phox -IR cells by about 30% (Fig. 5I and L).

### NAD(P)H Oxidase with OXA-induced Superoxide Production in Ventrolateral Medulla

**Figure 6:**
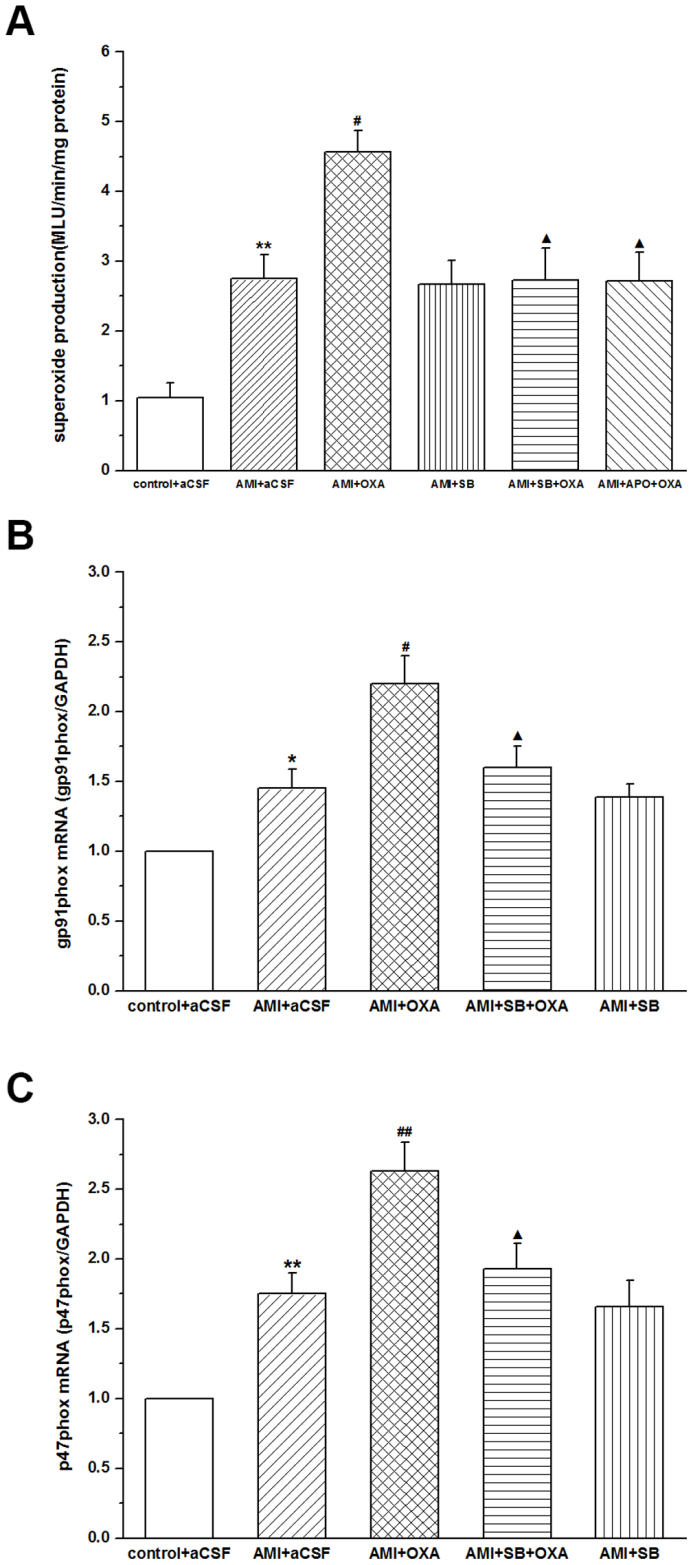
Measurements O_2_^−^ production and the mRNA of gp91phox and p47phox in RVLM. The results showing a significant increase in RVLM of OXA-treated rats than in that of the controls; SB-408124 (100pmol/100nl) or apocynin (APO, 100pmol/100nl) followed by OXA (100pmol) reducing the production in the RVLM; values as means ± S.E.M, n = 7; **P<0.01,*P<0.05 when compared with that of control+aCSF group; ^##^ *P*<0.01,^#^ *P*<0.05 when compared with AMI+aCSF group; ^▴^ *P*<0.05 when compared with AMI+OXA group.

O_2_^−^ production in RVLM was found to have a significant increase in AMI group when compared with that of the control (2.75±0.34 vs. 1.04±0.21; *P*<0.01) (Fig. 6A).

In AMI group, microinjection of OXA (1nmol) into the cerebral ventricle significantly increased O_2_^−^ production in RVLM compared with that of aCSF-treated rats (4.56±0.31 vs. 2.75±0.34; *P*<0.05). The effect of exogenous OXA on the generation of ROS was partially blocked by pre-microinjection of SB-408124 followed by OXA (4.56±0.31 vs. 2.73±0.46; *P*<0.05) (Fig. 6A).

To determine whether NAD(P)H oxidase was an enzymatic source of O_2_^−^, O_2_^−^ production was measured in the presence of APO, a NAD(P)H oxidase inhibitor, the results showing that O_2_^−^ production was significantly decreased in the absence of NAD(P)H oxidase in the rats (4.56±0.31 vs. 2.72±0.41; *P*<0.05) (Fig. 6A).

### Increased Expression of NAD(P)H Oxidase Subunits mRNA with OXA-treated in RVLM

Real time-PCR analysis showed the mRNA expression of NAD(P)H oxidase subunits in RVLM and significantly increased mRNA expression of gp91phox and p47phox in RVLM of AMI group in comparison with that of the control (Fig. 6B and 6C).

In AMI group, microinjection of OXA into the cerebral ventricle significantly increased gp91phox (2.2±0.2 vs. 1.45±0.14; *P*<0.05) and p47phox (2.63±0.21 vs. 1.75±0.15; *P*<0.01) mRNA in RVLM compared with that of aCSF-treated rats. The effect of exogenous OXA on the increase of gp91phox and p47phox mRNA was partially blocked by pre-microinjection of SB-408124 followed by OXA (gp91phox: 2.2±0.2 vs. 1.6±0.15; *P*<0.05; p47phox: 2.63±0.21 vs. 1.93±0.18; *P*<0.05) (Fig. 6B and 6C).

### NAD(P)H Oxidase-derived O2- in OXA-induced Cardiovascular Responses of AMI rats

**Figure 7:**
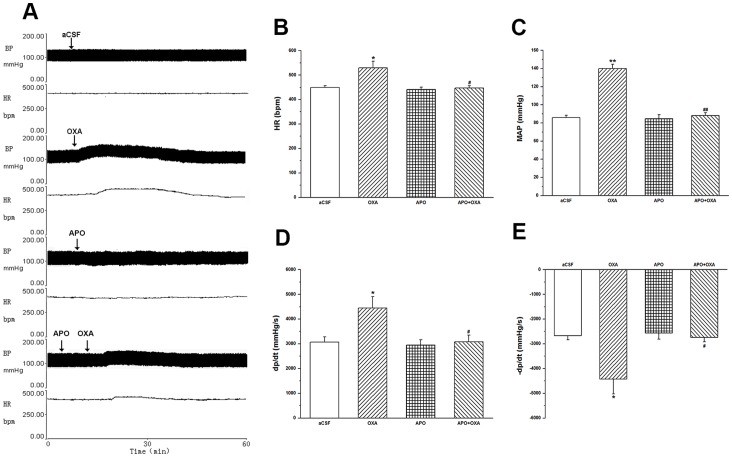
Effects of apocynin on OXA-induced cardiovascular responses in AMI rats. Exogenously administrated OXA into the RVLM evoking pressor and tachycardiac responses; apocynin (APO, 100 pmol/100 nl) followed by OXA (100 pmol) partly abolishing the pressor and tachycardiac responses of exogenously administrated OXA into the RVLM; values as means ± S.E.M, n = 7; *P<0.05, **P<0.01 when compared with aCSF group; ^#^ *P*<0.05,^ ##^ *P*<0.01 when compared with OXA group.

A single microinjection of APO into the unilateral RVLM of AMI rats slightly decreased MAP and HR, when compared with that of aCSF-treated rats, the results showing no statistical significance. Pre-microinjection of APO followed by OXA mostly blocked the left ventricle response induced by OXA alone: HR decreased from 530±27 to 447±9 bpm (*P*<0.05); MAP from 140±4.7 to 88±3.3 mmHg (*P*<0.01); and +LVdp/dtmax from 4446±467 to 3080±274 mmHg/s (*P*<0.05) and −LVdP/dtmax from −4428±602 to −2742±180 mmHg/s (*P*<0.05) (Fig. 7).

HRV power spectral analysis showed that in AMI group, APO resulted in an increase in HF (28.6±0.9 vs. 24.2±1.5; *P*<0.05) and a decrease in LF/HF ratio (0.39±0.02 vs. 0.59±0.02; *P*<0.01), when compared with that of the aCSF-treated group. Microinjection of APO followed by OXA partially blocked the changes of LF/HF ratio induced by OXA alone (0.68±0.02 vs. 0.93±0.05; *P*<0.01) (Table 2).

## Discussion

Ischemic heart diseases including myocardial infarction, angina pectoris and heart failure have been the leading cause of death world-wide. It has been reported that neurohumoral factors, especially autonomic nervous system, might play an important role in AMI [16], [17], [34]. Of the regulatory factors, OXA is increasingly concerned in the cardiac autonomic nervous system [7], [12], [14], [19]. OXA, as novel hypothalamic peptides, was reported to be initially found to be involved in the control of food intake in rats [6]. Studies showed that the peptide might play a role in other biological functions, such as arousal [35], [36], metabolism [37] and regulation of both respiratory [27], [38] and cardiovascular function [3], [4], [12], [13], [18]. In 1999, Shirasaka T et al. reported that intracerebroventricular administration of high dose (3.0nmol) of OXA rapidly increased MAP and HR in conscious and anesthetized rats [12]. Similarly, Samson WK et al. demonstrated that OXA stimulated a significant elevation in MAP in conscious, unrestrained rats [13]. But the cardiovascular regulatory actions of OXA were mostly observed in normal rats. Our previous study had showed that OXA in RVLM involved in the central regulation of cardiovascular activities in stress-induced hypertension rat [18]. In the current study, we employed AMI rat models to test the central regulation of cardiovascular activities in RVLM, and observed that the expression of OXA-IR neurons and the mRNA expression of PPO in AMI rats’ LHA were higher than that of the control group. Moreover, a decrease in HRV, coupling with hypertension and tachycardia, was observed when OXA (100pmol) was microinjected into the unilateral RVLM in AMI. The results indicated that OXA was involved in AMI.

The cardiac sympathetic nerve stimulation was reported to increase heart rate and the force of cardiac contraction, which could in turn attenuate cardiac function by increasing myocardial oxygen consumption [34], [39]. Therefore, sympatho-humoral activation following AMI was reported to be related to the extent of myocardial damage and associated with mortality and morbidity [16], [17]. It is well known that the RVLM is a critical neural region, which plays an important role in the generation and maintenance of sympathetic nerve activity [40], [41]. Though OXA-containing neurons are located in LHA, their terminals and receptors are widely distributed in many brain regions including the RVLM [7]–[9]. And it has been reported that OXA induced hypertension and tachycardia were ascribed to an increase in sympathetic vasomotor tone mediated by RVLM [11], [18]. Microinjection of OXA to the RVLM area can elevate blood pressure and HR in anesthetized and awake rats [11], [19]. These studies have provided pharmacological evidence that OXA could activate the cardiovascular related neurons in RVLM, which leads to an increase of medullary sympathetic output. Furthermore, Dun NJ et al. found that OXA could depolarize directly neurons in the RVLM [42]. Thus we explored the cardiovascular regulatory actions of OXA upon microinjection to the RVLM in AMI rats.

In the current study, the ratio of LF and HF component of HRV was analyzed to evaluate cardiac sympathetic activity. Our results showed that LF/HF ratio, which was reported to mean more cardiac sympathetic activity [43], was increased in AMI rats when compared with that of the control group. In addition, the concentrations of norepinephrine and epinephrine in the serum increased significantly in AMI rats (Figure S2). These results indicated that high sympathetic tone was one of the characteristics in AMI rats. Microinjection of SB-408124 or TCS OX2 29 decreased the LF/HF ratio of AMI rats. Moreover, both the antagonists partly decreased the LF/HF ratio of AMI rats evoked by OXA exogenously-administrated into RVLM.The effect of SB-408124 was found to be greater than that of TCS OX2 29, which suggested that OX_1_R played a more important role in the changes. Therefore, we selected SB-408124 to further verify the effect of OXA on cardiac dysfunction in AMI. Consequently, it was found out that the higher sympathetic tone in AMI was caused by the higher level of OXA.

Under various pathophysiological conditions of patients with heart disease, such as ischemic heart disease and heart failure, the LV dysfunction, including diastolic and systolic dysfunction, would emerge [44]. +LVdp/dtmax and −LVdp/dtmax, applied to the evaluation of the LV function, have been widely used in the clinical setting. In the current study, we observed that the absolute value of +LVdp/dtmax and −LVdp/dtmax, coupling with HR and MAP, decreased in AMI rats when compared with that in the controls, which indicated that impaired myocardium could lead to LV diastolic and systolic dysfunction. Microinjection of SB-408124 or TCS OX2 29 alone into the RVLM of AMI rats produced no obvious effect on MAP and HR. However, that of SB-408124 or TCS OX2 29 into the RVLM almost abolished the cardiovascular responses evoked by OXA exogenously-administrated into the RVLM. Similarly, Kazuyoshi Hirota et al. reported that SB-334867 could reverse the responses evoked by OXA both *in vivo* and *in vitro*, and when administrated SB-334867 alone, did not significantly change baseline hemodynamic variables [45]. It is worth noting that the absolute value of +LVdp/dtmax and −LVdp/dtmax increased within 60min after OXA exogenously-administrated into RVLM. The reason can be that OXA (100pmol) exogenously-administrated into RVLM of AMI rats might arouse a transient increase of systolic and diastolic motion velocities and force. It is well known that the over excitation of sympathetic nervous system can attenuate cardiac function when myocardial infarction occurs, but OXA (100pmol), exogenously administrated into RVLM of AMI tats, can arouse a higher sympathetic tone than when there is only the presence of myocardial infarction, so that the cardiac function seems to have been improved. But we reasoned that myocardial damage and cardiac function deteriorated further with the efficacy of exogenous OXA wearing off. This needs further investigations, for the current study did not focus on the changes of cardiac function indexes 60min latter after the exogenous administration of OXA into RVLM.

Though OXA was found to activate the cardiac sympathetic neurons in RVLM, the mechanism was not clear. The relationship between the central ROS and sympathetic nerve activity has proved an immensely rewarding focus for research, a large number of studies demonstrating that central ROS would be involved in the cardiovascular responses via sympathoexcitory pathay [21]–[24], [42], [46]–[48]. In RVLM, ROS might be involved in the central sympathoexcitation of hypertension [47] and myocardial infarction-induced heart failure [24], and NAD(P)H oxidase may play an important role in generation of superoxide anion (O_2_^−^) [23], [24], [46]. In the current study, we observed that the OX_1_R-IR cells were co-localized with NAD(P)H oxidative subunit immunoreactive cells (both gp91phox and p47phox) in RVLM, and that O_2_^−^ production was increased by centrally administered OXA. Real-time PCR analysis showed that microinjection of OXA into the cerebral ventricle significantly increased the mRNA expression of gp91phox and p47phox in RVLM when compared with that of aCSF-treated and OXA plus SB-408124-treated rats in RVLM. Furthermore, we explored the effect of NAD(P)H oxidase p47phox subunit inhibitor, apocynin (APO), on the cardiovascular response evoked by centrally administered OXA. The results showed that microinjection of APO alone into the RVLM of AMI rats produced no obvious effect on O_2_^−^ production and OXA-induced cardiovascular responses in AMI group; however, microinjection of APO into the RVLM almost abolished the cardiovascular responses evoked by OXA administrated exogenously into the RVLM. These results indicated that the cardiovascular responses of OXA in RVLM of AMI was mediated by NAD(P)H oxidase-derived ROS, which played an important part in the signaling pathway mediating cardiovascular and sympathetic nerve responses to the central administration of OXA.

We concluded that OXA in the RVLM might participate in the central regulation of cardiovascular activities via the increased sympathetic activity in AMI. The OXA-mediated cardiovascular mechanism as follows: OXA-IR neurons in LHA, projecting to the cardiac sympathetic center in RVLM to bind with their receptors including OX_1_R and OX_2_R, thereby increasing the superoxide anion (O_2_^−^) via NAD (P) H oxidation-reduction system and activating the sympathetic nervous system, which then regulated the cardiovascular activities. The findings can be valuable for future investigations in OXA-mediated cardiovascular mechanism.

In limitation, we used the indirect indices of HRV to assess the vagal/sympathetic nerve activity; therefore, the direct magnitude of sympathetic drive and its relationship with OXA-OXR receptor over long-term cardiac function post-acute myocardial infarction merit further investigations.

## Supporting Information

Figure S1 **Identified microinjection sites in RVLM using 1% Neutral Red staining.** Scale bar = 1mm.
(TIF)

Figure S2 Plasma epinephrine (E) and norepinephrine (NE) concentration in control and AMI rats immunoenzymatically determined; values as mean+S.E.M (n = 6),^ *^ ***P*** **<0.05 when compared with the control.**
(TIF)

Figure S3 Effects of SB-408124, or TCS OX2 29 or apocynin on OXA-induced cardiovascular responses in the controls; values as means ± S.E.M, n = 7; * ***P*** <0.05, ** ***P*** **<0.01 when compared with aCSF group; ^#^** ***P*** **<0.05, ^##^** ***P*** **<0.01 when compared with OXA group.**
(TIF)
