# Supplementary figures and images for: The Effect of Orexin-A on Cardiac Dysfunction Mediated by NADPH Oxidase-Derived Superoxide Anion in Ventrolateral Medulla
Source: PLoS One. 2013 Jul 26;8(7):e69840. doi: 10.1371/journal.pone.0069840 (PMC3724905; doi:10.1371/journal.pone.0069840)

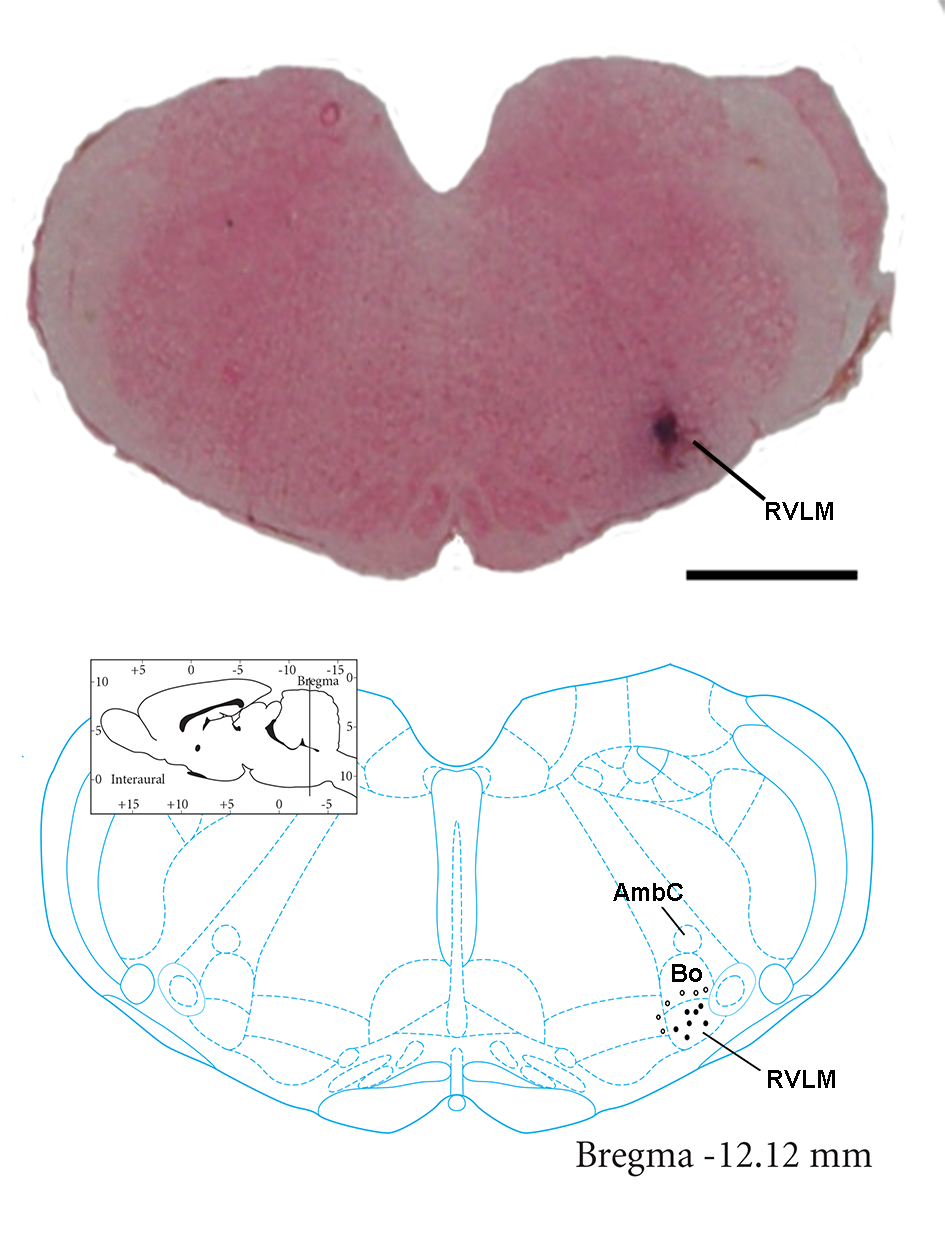

Supplement: Figure S1 — Identified microinjection sites in RVLM using 1% Neutral Red staining. Scale bar = 1mm. (TIF) [file pone.0069840.s001.tif]

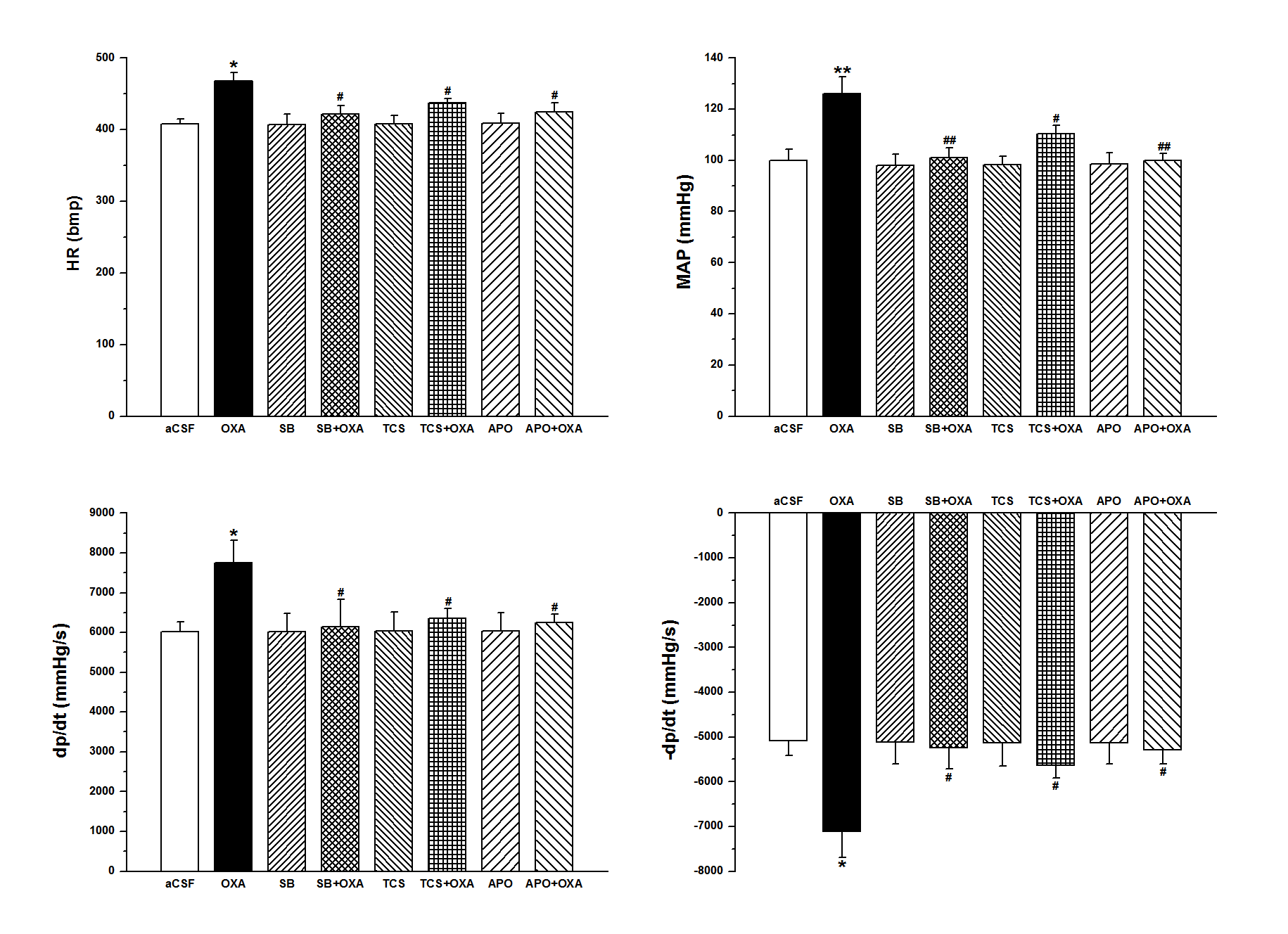

Supplement: Figure S3 — Effects of SB-408124, or TCS OX2 29 or apocynin on OXA-induced cardiovascular responses in the controls; values as means ± S.E.M, n = 7; * P <0.05, ** P <0.01 when compared with aCSF group; # P <0.05, ## P <0.01 when compared with OXA group. (TIF) [file pone.0069840.s003.tif]
